# An advanced approach for the electrical responses of discrete fractional-order biophysical neural network models and their dynamical responses

**DOI:** 10.1038/s41598-023-45227-8

**Published:** 2023-10-24

**Authors:** Yu-Ming Chu, Taher Alzahrani, Saima Rashid, Waleed Rashidah, Shafiq ur Rehman, Mohammad Alkhatib

**Affiliations:** 1 School of Science, Hunan City University, Yiyang, 413000 People’s Republic of China; 2 Information Systems Department, College of Computer and Information Sciences, Imam Mohammad Ibn Saud Islamic University (IMSIU), Riyadh, 11432 Saudi Arabia; 3 Department of Mathematics, Government College University, Faisalabad, 38000 Pakistan; 4 Department of Computer Science and Mathematics, Lebanese American University, Beirut, 1401 Lebanon; 5 Computer Science Department, College of Computer and information Sciences, Imam Mohammad Ibn Saud Islamic University (IMSIU), Riyadh, 11432 Saudi Arabia

**Keywords:** Biophysics, Neuroscience, Mathematics and computing

## Abstract

The multiple activities of neurons frequently generate several spiking-bursting variations observed within the neurological mechanism. We show that a discrete fractional-order activated nerve cell framework incorporating a Caputo-type fractional difference operator can be used to investigate the impacts of complex interactions on the surge-empowering capabilities noticed within our findings. The relevance of this expansion is based on the model’s structure as well as the commensurate and incommensurate fractional-orders, which take kernel and inherited characteristics into account. We begin by providing data regarding the fluctuations in electronic operations using the fractional exponent. We investigate two-dimensional Morris–Lecar neuronal cell frameworks via spiked and saturated attributes, as well as mixed-mode oscillations and mixed-mode bursting oscillations of a decoupled fractional-order neuronal cell. The investigation proceeds by using a three-dimensional slow-fast Morris–Lecar simulation within the fractional context. The proposed method determines a method for describing multiple parallels within fractional and integer-order behaviour. We examine distinctive attribute environments where inactive status develops in detached neural networks using stability and bifurcation assessment. We demonstrate features that are in accordance with the analysis’s findings. The Erdös–Rényi connection of asynchronization transformed neural networks (periodic and actionable) is subsequently assembled and paired via membranes that are under pressure. It is capable of generating multifaceted launching processes in which dormant neural networks begin to come under scrutiny. Additionally, we demonstrated that boosting connections can cause classification synchronization, allowing network devices to activate in conjunction in the future. We construct a reduced-order simulation constructed around clustering synchronisation that may represent the operations that comprise the whole system. Our findings indicate the influence of fractional-order is dependent on connections between neurons and the system’s stored evidence. Moreover, the processes capture the consequences of fractional derivatives on surge regularity modification and enhance delays that happen across numerous time frames in neural processing.

## Introduction

In the discipline of dynamic investigation, neural systems have been acknowledged as being among the increasingly essential dynamic representations. The main justifications behind these systems’ importance are their framework and simultaneous execution capability. Because of their multiple uses in numerous disciplines, including optimization, memory consolidation, confidentiality of data, image recognition, and many more, neuronal network theory has received a lot of consideration from scientists over the past few decades^1–3^. The fluctuations of the neural network’s communications are being extensively studied in mathematical physics and technology. The aforementioned models’ evaluation revealed multiple broader characteristics, including bifurcations, constraint cycles, and chaotic behaviour^4,5^. Despite the many research investigations that have been conducted to examine the evolving behaviours of continuous-time structures, discrete-time methods and mechanisms have attracted much fewer resources. Discrete-time frameworks have distinguished evolving characteristics, and they can be employed to symbolize a wide range of beneficial complications within the reality of life^6,7^. Because of such features, research into independent neural network mechanisms is critical and has culminated in major discoveries in physical science, mathematical concepts, and numerous other disciplines^8,9^.

Discrete fractional calculus encounters have sparked the interest of many scientists over the past 20 years, owing to their significance for disciplines as diverse as computer application, photo encoding, and encrypted interaction. A plethora of publications on this popular subject have recently been published, with the authors offering an assortment of discrete-time fractional operators, durability assessments, and numerous mathematical findings^10–12^. Alsharidi et al.^13^ investigated a short-memory discrete fractional difference equation wind turbine model using a Caputo-type fractional difference operator. Al-Qurashi et al.^14^ expounded the complexity analysis and discrete fractional difference implementation of the Hindmarsh-Rose neuron system. Chu et al.^15^ derived the new configurations of the fuzzy fractional differential Boussinesq model with application in ocean engineering. Wu and Baleanu^16^ established their inaugural research, which concerned the modelling of the fractional chaotic map employing the Caputo difference operator and researching its turbulent features. These have led to the proposal of more commensurate fractional discrete chaotic systems, such as^17^, and more incommensurate fractional discrete chaotic systems, such as^18^, along with a variety of control strategies and synchronization schemes between different fractional chaotic maps, such as^19^.

Numerous researchers have been investigating the oscillatory patterns of fractional continuous-time neural network frameworks due to their prospective functions in various disciplines such as pattern identification, computational optimization, connected retention, and data processing^20,21^. However, when implementing continuous-time structures for computerized processing, exploration, or modelling, it is crucial to isolate continuous-time structures regardless of whether they ought to be implemented for technological device estimation, exploration, or modelling. Discrete-time neural networks have been developed and are used in an extensive spectrum of domains^22,23^. Mohamad and Gopalsamy^19^ discussed the exponential stability of continuous and discrete-time cellular neural networks with delays. As a result, an examination of the functioning of discrete-time neural network is required. A while ago, a couple of studies were published that investigated the evolving dynamics of fractional discrete-time modelling in neural network^23–24^. Weinberg^25^ addressed how membrane conductive storage affects spikes in nerve cells characterized by the fractional-order Hodgkin-Huxley neural network framework. Lundstrom et al.^26^ contemplated the fractional operators by neurons in pyramids of the neocortex. Huang et al.^27^ expounded the new fractional discrete-time neural network based on the variable order. Allehiany^28^ presented the chaotic regulatory and fractional-order dynamics of neural network under electromagnetic radiation. A significant amount of the previously stated discrete-time neural network studies focus on frameworks with corresponding or incommensurate fractional-orders; however, currently, as far as we comprehend, only a handful of improvements have been published that examine the exciting practises of fractional-order discrete-time neural network frameworks^29–31^. As a consequence, studying the unpredictable behaviour of neural network frameworks driven by fractional differences with commensurate and incommensurate values, in addition to their synchronization and oversight, is an appealing topic.

Influenced by the previous debate, we aim to investigate and analyze the fluctuating patterns of the intriguing two- and three-dimensional discrete fractional-order Morris-Lecar neural network paradigm using electrically powered operations of independent Morris-Lecar firing neural network regarding Groups **i** and **ii** activities and slow-fast Morris-Lecar neural network^32,33^ concerning its presence design employing commensurate and incommensurate fractional-orders in the present article. The key elements of this discrete fractional-order system will be investigated by implementing hypothetical and computational assessments. Take into account a conductance-dependent simulation that investigates the inherent behaviour underpinning fractional-order differences when investigating these terminating features and transformations. Prior studies examined different periods of spiked remarks based on varying setting regimes; despite this, discrete fractional-order can also evaluate broad-sack reactions^34^. The Morris–Lecar frameworks are used because of their respective positions in various reactions, which range from spikes to ruptures. In the parametric forum of the Morris-Lecar approach, it includes two regimes: dominant spiked and swift spiked behaviours. Furthermore, the representation has been transformed into a three-dimensional equivalent in which the generated electricity, $\Im$, remains steady but fluctuates with energy. We take the discrete fractional-order technique to be the most significant method in the framework, considering that it modifies gradually, spike adjustments happen, and mixed mode oscillations and mutated monarch butterfly optimizations are observed. It constitutes one of the particularly fascinating neurological variations that arise from electrical activity^35^. Mixed mode oscillations indicate the reversed pathways of tiny and massive intensity resonances^36^. This makes the structure captivating, and its outcome supplies exciting and potentially advantageous uses in an adaptive structure. The resulting appearance of mutated monarch butterfly optimizations results in the creation of an elevated incorporation process. Previously, it was discovered that mixed mode oscillations examined the evolving and neurological behaviour of manoeuvring or breathing^37^. Calcium signalling and electrocardiac applications were both affected^38^. Krupa et al.^39^ investigated the process of mixed mode oscillation fluctuations in the mammal cerebral cortex using a two-dimensional representation of dopamine signalling. We additionally glance at how electronic connections affect an assortment of cells that are, in two ways, inactive or periodic. In this case, the synapses of neural networks are considered to be associated via the pathways of the Erdös-Rényi structure. To identify all components in the structure, the interaction causes intricate terminating operations that involve repeated overflowing or surge regularity adaptation mechanisms. Depending on the discovered asymptotic occurrences, a diminished-order emulate is established that can generate the whole system’s actions.

Employing the fractional exponent, we discovered commensurate and incommensurate differences in the features of neurologically effective behaviour. Fractional-order power interactions may drastically impact the spikes in the characteristics of various unattached neuron models^40,41^. Practical characteristics are capable of rendering the mathematical framework more responsive to neuronal activity interactions, especially in the network’s prospective collaborative behaviour, in which previous dynamic behaviour could impact current conditions.

The remaining content of this article is structured as follows: The qualitative description of the discrete fractional-order neural network system in terms of the electrical nature of independent Morris–Lecar spike neurons via category **i** and **ii** activities is presented in a detailed manner. Furthermore, the slow-fast Morris–Lecar cells employing the Caputo difference expression are presented in Section 2. In Section 3, we investigate the framework’s fundamentally evolving characteristics using computation and analytical techniques of the two-dimensional discrete fractional-order Morris–Lecar neural network model. Section 4 demonstrates the detailed analysis of discrete fractional-order Morris–Lecar neural networks with commensurate and incommensurate fractional-orders. Moreover, the various oscillatory responses are presented with detailed simulation techniques. To stabilize and synchronize the unstable pathways of the suggested fractional discrete-time neural network approach, we put forward responsive processors. Ultimately, Section 6 presents the overall paper’s ending.

## Mathematical model

In this part, we indicate the fractional-order conductance-based simulation and discuss the presence of multiple neural characteristics^42^. We determine a specific setting structure that enables terminating characteristics via fractional exponent modifications. We investigate the two- and three-dimensional Morris–Lecar frameworks via specific settings and transmit interactions for producing broad surges employing fractional-order behaviour. We chose one of these frameworks to distinguish the influence of fractional formulations on the system’s dynamic practises. Morris and Lecar^32^ suggested a simple mathematical design that described fluctuations in the barnacle’s enormous muscle fibre, which included the cellular electricity formula alongside immediately apparent calcium up-to-date stimulation and a supplementary restoration formula explaining slow potassium energy initialization. In the fractional-order sense, the two-dimensional Morris–Lecar system is explained as follows:

1 $$\begin{aligned} {\left\{ \begin{array}{ll} {\mathcal {C}}\frac{d^{\vartheta }{u_{1}}}{d\xi ^{\vartheta }}=-1/2{\mathcal {W}}_{Ca}({u_{1}}-{\mathcal {F}}_{Ca})\left( (1+\tanh (\sigma -{\mathcal {F}}_{1}))/{\mathcal {F}}_{2}\right) -{u_{2}} {\mathcal {W}}_{{\mathcal {K}}}({u_{1}}-{\mathcal {F}}_{{\mathcal {K}}})\\ \qquad \qquad -{\mathcal {W}}_{{\mathcal {L}}}({u_{1}}-{\mathcal {F}}_{{\mathcal {L}}})+\Im =\hbar _{1}({u_{1}},{u_{2}}),\\ \frac{d^{\vartheta }{u_{2}}}{d\xi ^{\vartheta }}=\varphi \cosh ({u_{1}}-{\mathcal {F}}_{3})/2{\mathcal {F}}_{4}\left( 1/2(1+\tanh (({u_{1}}-{\mathcal {F}}_{3})/{\mathcal {F}}_{4}))-{u_{2}}\right) =\hbar _{2}({u_{1}},{u_{2}}).\end{array}\right. } \end{aligned}$$

A voltage-gated $Ca^{2+}$ energy, an induced converter ${\mathcal {K}}^{+}$ energy, and a leaky energy are all part of the biophysically stimulated activated approach. The membrane power interactions are measured by ${u_{1}}$, and the stimulation attribute of ${\mathcal {K}}^{+}$ charge activity is ${u_{2}}$. The highest conductivity operates to $Ca^{+},\,{\mathcal {K}}^{+}$ and release flows are indicated by the factors ${\mathcal {W}}_{Ca},\,{\mathcal {W}}_{{\mathcal {K}}}$ and ${\mathcal {W}}_{{\mathcal {L}}}$, respectively. The transformations of prospective to various ionised energy performs are ${\mathcal {F}}_{Ca},\,{\mathcal {F}}_{{\mathcal {K}}}$ and ${\mathcal {F}}_{{\mathcal {L}}}$. The protective layer capaciousness is measured by ${\mathcal {C}}$ symbolizes the ambient temperature developing rate for revealing the ${\mathcal {K}}^{+}$ pathway. The settings for ${\mathcal {F}}_{1},\,{\mathcal {F}}_{2},\,{\mathcal {F}}_{3}$ and ${\mathcal {F}}_{4}$ have constant positive values. The letter $\Im$ denotes the substance that was used stimulus. We want to take consideration of the influence of different inserted energy stimuli on the fractional-order framework based on $\vartheta \in (0,1].$

### Diverse dynamical aspects

Initially, suppose the neuron is just starting to fire and is generating spikes as the regulation variable advances cautiously. Slow-fast interactions^42^ can be computationally represented as

2 $$\begin{aligned}{} & {} \dot{{\textbf{y}}}(\xi )=\Theta ({\textbf{y}},{\textbf{z}}),\nonumber \\{} & {} \dot{{\textbf{z}}}(\xi )=\zeta {\textbf{h}}({\textbf{y}},{\textbf{z}}), \end{aligned}$$

where $\dot{{\textbf{y}}}(\xi )=\Theta ({\textbf{y}},{\textbf{z}})$ indicates fast spiking and $\dot{{\textbf{z}}}(\xi )=\zeta {\textbf{h}}({\textbf{y}},{\textbf{z}})$ represents the slow modulation. The rapidly changing parameters are represented by ${\textbf{y}}\in {\mathbb {R}}^{{\textbf{r}}}$ and the slow factors are represented by ${\textbf{z}}\in {\mathbb {R}}^{{\textbf{s}}}$, with $0<\zeta \ll 1$ assessing the duration of the split parameter estimation.

The slow-fast three-dimensional Morris–Lecar model is represented by a corresponding structure of ordinary differential equations, where $({u_{1}},{u_{2}})$ indicate the fast component and ${u_{3}}$ the slow variable. The fractional-order improved three-dimensional Morris–Lecar model^32,42^ is shown as follows:

3 $$\begin{aligned} {\left\{ \begin{array}{ll} {\mathcal {C}}\frac{d^{\vartheta }{u_{1}}}{d\xi ^{\vartheta }}=-1/2{\mathcal {W}}_{C_{a_{1}}}({u_{1}}-1)\left( (1+\tanh ({u_{1}}-{\mathcal {F}}_{1}))/{\mathcal {F}}_{2}\right) -{u_{2}} {\mathcal {W}}_{{\mathcal {K}}}({u_{1}}-{\mathcal {F}}_{{\mathcal {K}}})-{\mathcal {W}}_{{\mathcal {L}}}({u_{1}}-{\mathcal {F}}_{{\mathcal {L}}})\\ \qquad \qquad +\Im ({u_{3}})=\Theta _{1}({u_{1}},{u_{2}},{u_{3}}),\\ \frac{d^{\vartheta }{u_{2}}}{d\xi ^{\vartheta }}=\varphi \cosh \left( ({u_{1}}-{\mathcal {F}}_{2})/2{\mathcal {F}}_{4}\right) \left( 1/2(1+\tanh \left( {u_{1}}-{\mathcal {F}}_{3}\right) /{\mathcal {F}}_{4})-{u_{2}}\right) =\Theta _{2}({u_{1}},{u_{2}},{u_{3}}),\\ \frac{d^{\vartheta }{u_{3}}}{d\xi ^{\vartheta }}=\theta ({\mathcal {F}}_{0}+{u_{1}})=\Theta _{3}({u_{1}},{u_{2}},{u_{3}}). \end{array}\right. } \end{aligned}$$

The framework has a few additional features. Externally administered energy, which implies index-law interactions in the fractional framework and illustrates the memory impact of the cellular within the voltage^32,42^, is the mechanism factor ${u_{3}}$. The hyperbolic mappings’ settings ${\mathcal {F}}_{1},\,{\mathcal {F}}_{2},\,{\mathcal {F}}_{3}$ and ${\mathcal {F}}_{4}$ are effectively chosen to demonstrate how they are capable of reaching their fixed points instantly. The specified worth value $\theta$ is smaller than one, i.e., $\theta \in (0,1)$, and it determines the length of time scale ratio between resonances and modification. To identify this kind of feedback, Lundstrom et al^26^ discovered that neuronal pyramids are able to function as fractional differentiation of the challenge intensity envelope. To determine the initial differentiation of a mapping, differentiate twice by implementing the fractional-order derivative of $\vartheta =1/2$ which leads to the first derivative^43,44^. It adjustments the reaction using a decomposing time delay that is proportional to $\vartheta .$

To identify all simulated outcomes, the attribute distinguishes are (for (1), see^33,42^):

Group **i**: ${\mathcal {C}}=20.0,\,{\mathcal {W}}_{Ca}=4.0,\,{\mathcal {W}}_{{\mathcal {K}}}=8.0,\,{\mathcal {W}}_{{\mathcal {L}}}=2.0,\,{\mathcal {F}}_{Ca}=120.0,\,{\mathcal {F}}_{{\mathcal {K}}}=-84.0,\,{\mathcal {F}}_{{\mathcal {L}}}=-60.0,\,{\mathcal {F}}_{1}=-1.20,\,{\mathcal {F}}_{2}=18.0,\,{\mathcal {F}}_{3}=12.0,\,{\mathcal {F}}_{4}=17.40,\,\varphi =0.0670$ (for the excited cell simulation of category ${\textbf {i}}$) with various $\Im .$ Group **ii**: $\Im =40.$ Group **ii**: $\Im =45$ and for the group **ii** membrane framework, the specifications are actually identical mentioned previously, aside for Group **iii**: ${\mathcal {W}}_{Ca}=4.4,\,{\mathcal {F}}_{3}=2.0,\,{\mathcal {F}}_{4}=30,\,\varphi =0.04$ and $\Im =100.$ To investigate the functioning of systems, we initially examine neutral states and then bifurcation. afterwards that, we employ the subsequent variable distinguishes for interacting with structures (1) and its improved forms, alongside $\Im ({u_{3}})=0.08-0.03{u_{3}}$ and ${\mathcal {C}}=1$ for attribute initiates **i**, **ii** and **iii**, respectively.

Group **i**: $${\mathcal {W}}_{Ca}=0.9,\,{\mathcal {W}}_{{\mathcal {K}}}=2,\,{\mathcal {W}}_{{\mathcal {L}}}=1/2,\,{\mathcal {F}}_{Ca}=1,\,{\mathcal {F}}_{{\mathcal {K}}}=-0.7,\,{\mathcal {F}}_{{\mathcal {L}}}=-1/2,\,{\mathcal {F}}_{1}=-0.01,\,{\mathcal {F}}_{2}=0.15,\,{\mathcal {F}}_{3}({u_{3}})=(0.08-{u_{3}}),\,{\mathcal {F}}_{4}=0.04,\,\varphi =0.033,\,{\mathcal {F}}_{0}=0.22,\,\theta =0.003.$$

Group **ii**: $${\mathcal {W}}_{Ca}=1.36,\,{\mathcal {W}}_{{\mathcal {K}}}=2,\,{\mathcal {W}}_{{\mathcal {L}}}=1/2,\,{\mathcal {F}}_{Ca}=1,\,{\mathcal {F}}_{{\mathcal {K}}}=-0.7,\,{\mathcal {F}}_{{\mathcal {L}}}=-1/2,\,{\mathcal {F}}_{1}=-0.01,\,{\mathcal {F}}_{2}=0.15,\,{\mathcal {F}}_{3}({u_{3}})=(0.08-{u_{3}}),\,{\mathcal {F}}_{4}=0.16,\,\varphi =0.033,\,{\mathcal {F}}_{0}=0.1,\,\theta =0.003.$$

Group **iii**: ${\mathcal {W}}_{Ca}=0.9,\,{\mathcal {W}}_{{\mathcal {K}}}=2,\,{\mathcal {W}}_{{\mathcal {L}}}=1/2,\,{\mathcal {F}}_{Ca}=1,\,{\mathcal {F}}_{{\mathcal {K}}}=-0.7,\,{\mathcal {F}}_{{\mathcal {L}}}=-1/2,\,{\mathcal {F}}_{1}=-0.01,\,{\mathcal {F}}_{2}=0.15,\,{\mathcal {F}}_{3}({u_{3}})=(0.08-{u_{3}}),\,{\mathcal {F}}_{4}=0.05,\,\varphi =0.033,\,{\mathcal {F}}_{0}=0.,\,\theta =0.005.$ To investigate the fractional-order Morris–Lecar framework, we employ the well-known description of the fractional difference, namely the Caputo-type fractional difference operator. The commensurate fractional-order framework with the fractional exponent $\vartheta \in (0,1)$ is defined as follows:

4 $$\begin{aligned} {\left\{ \begin{array}{ll} {\mathcal {C}}\,^{c}{\textbf{D}}^{q_{1}}{u_{1}}(\xi )=-1/2{\mathcal {W}}_{C_{a_{1}}}({u_{1}}-1)\left( (1+\tanh ({u_{1}}-{\mathcal {F}}_{1}))/{\mathcal {F}}_{2}\right) -{u_{2}} {\mathcal {W}}_{{\mathcal {K}}}({u_{1}}-{\mathcal {F}}_{{\mathcal {K}}})-{\mathcal {W}}_{{\mathcal {L}}}({u_{1}}-{\mathcal {F}}_{{\mathcal {L}}})+\Im ({u_{3}}),\\ \,^{c}{\textbf{D}}^{q_{2}}{u_{2}}(\xi )=\varphi \cosh \left( ({u_{1}}-{\mathcal {F}}_{2})/2{\mathcal {F}}_{4}\right) \left( 1/2(1+\tanh \left( {u_{1}}-{\mathcal {F}}_{3}\right) /{\mathcal {F}}_{4})-{u_{2}}\right) ,\\ \,^{c}{\textbf{D}}^{q_{3}}{u_{3}}(\xi )=\theta ({\mathcal {F}}_{0}+{u_{1}}), \end{array}\right. } \end{aligned}$$

where $q_{1},\,q_{2}$ and $q_{3}$ are the fractional-order such that $q{\iota }\in (0,1).$ $\,^{c}{\textbf{D}}^{q_{1}}$ is the Caputo fractional derivative which can be evaluated by the following formula:

$$\begin{aligned} \,^{c}{\textbf{D}}^{q_{1}}{\mathcal {F}}(\xi )=\frac{1}{\Gamma (1-q_{\iota })}\int \limits _{a_{1}}^{\xi }(\xi -\chi )^{-q_{\iota }}{\mathcal {F}}(\chi )d\chi . \end{aligned}$$

It is emphasized that when $q_{1}=q_{2}=q_{3},$ a commensurate fractional-order model is developed; otherwise, an incommensurate fractional-order model is formed.

## Preliminaries on discrete fractional calculus

Prior to defining our fractional discrete framework, we will go over certain crucial terms and mathematical principles of discrete fractional calculus.

### Definition 3.1

(^10^) Assume that there is a time scale ${\mathbb {N}}_{{\textbf{a}}}=\left\{ {\textbf{a}},{\textbf{a}}+1,{\textbf{a}}+2,\ldots \right\} , \,{\textbf{a}}\in {\mathbb {R}}.$ The fractional sum of order $\vartheta$ for a mapping ${\mathcal {F}}$ can be defined as

5 $$\begin{aligned} \Delta _{{\textbf{a}}}^{-\vartheta }{\mathcal {F}}(\xi )=\frac{1}{\Gamma (\vartheta )}\sum \limits _{\Upsilon =0}^{\xi -{\textbf{a}}}(\xi -1-\Upsilon )^{(\vartheta -1)}{\mathcal {F}}(\Upsilon ),\,\,\forall {\textbf{a}}>0,\,\xi \in {\mathbb {N}}_{{\textbf{a}}+\vartheta }. \end{aligned}$$

### Definition 3.2

(^11^) The $\vartheta$-Caputo fractional difference operator is described as follows

6 $$\begin{aligned} \,^{c}\Delta _{{\textbf{a}}}^{\vartheta }{\mathcal {F}}(\xi )= & {} \Delta _{{\textbf{a}}}^{-({\textbf{q}}-\vartheta )\Delta ^{{\textbf{q}}}}{\mathcal {F}}(\xi )\nonumber \\= & {} \frac{1}{\Gamma ({\textbf{q}}-\vartheta )}\sum \limits _{\Upsilon =0}^{\xi -({\textbf{q}}-\vartheta )}(\xi -1-\Upsilon )^{({\textbf{q}}-\vartheta -1)}\Delta ^{{\textbf{q}}}{\mathcal {F}}(\Upsilon ), \end{aligned}$$

where $\xi \in {\mathbb {N}}_{{\textbf{a}}+{\textbf{q}}-\vartheta },\,\vartheta \notin {\mathbb {N}}$ and ${\textbf{q}}=\lceil \vartheta \rceil +1.$ $\Delta ^{{\textbf{q}}}{\mathcal {F}}(\Upsilon )$ and $(\xi -1-\Upsilon )^{({\textbf{q}}-\vartheta -1)}$ represents the $m^{th}$ integer difference operator and falling factorial mapping, respectively, indicated as

7 $$\begin{aligned} (\xi -1-\Upsilon )^{({\textbf{q}}-\vartheta -1)}=\frac{\Gamma (\xi -\Upsilon )}{\Gamma (\xi -\Upsilon -{\textbf{q}}+\vartheta +1)} \end{aligned}$$

and

8 $$\begin{aligned} \Delta ^{{\textbf{q}}}{\mathcal {F}}(\xi )= & {} \Delta \left( \Delta ^{{\textbf{q}}-1}{\mathcal {F}}(\xi )\right) \nonumber \\= & {} \sum \limits _{\chi =0}^{{\textbf{q}}}\begin{pmatrix} {\textbf{q}}\\ \chi \end{pmatrix}(-1)^{{\textbf{q}}-\chi }{\mathcal {F}}(\xi +\chi ),\,\,\xi \in {\mathbb {N}}_{{\textbf{a}}}. \end{aligned}$$

Currently, we require the resulting hypothesis^45^ to figure out the stability manipulations to stay on the critical highlights of a fractional discrete mechanism with commensurate fractional-order characteristics:

### Theorem 3.1

(^44^) *Assume that there is a fractional-order* $\hbar (\xi )=\left( {\hbar }_{1}(\xi ),\ldots ,{\hbar }_{{\textbf{q}}}(\xi )\right) ^{{\textbf{T}}},\,\vartheta \in (0,1)$ *and* ${\mathcal {W}}\in {\mathbb {R}}^{{\textbf{q}}\times {\textbf{q}}},$ *the zero steady states of the commensurate discrete fractional-order framework*

9 $$\begin{aligned} \,^{c}\Delta _{{\textbf{a}}}^{\vartheta }{\hbar }(\xi )={\mathcal {W}}{\hbar }(\xi -1+\vartheta ) \end{aligned}$$

*for all* $\xi \in {\mathbb {N}}_{{\textbf{a}}+1-\vartheta }$ *is asymptotically stable if* $\wp _{\lambda }\in \left\{ w_{1}\in \tilde{{\mathcal {C}}}:\big \vert w_{1}\big \vert \le 2\left( 2\cos \frac{\big \vert \arg w_{1}\big \vert -\pi }{2-\vartheta }\right) ^{\vartheta }\,\,and\,\,\big \vert \arg w_{1}\big \vert \ge \frac{\vartheta \pi }{2}\right\} ,$ *where* $\wp _{\lambda }$ *indicates the eigenvalues of the matrix* ${\mathcal {W}}.$

However, the reliability and robustness concept of the dynamic fractional-order incommensurate framework is formulated as follows:

### Theorem 3.2

(^45^) *Assume the system*

10 $$\begin{aligned} {\left\{ \begin{array}{ll} \,^{c}\Delta _{{\textbf{a}}}^{\vartheta _{1}}{{\textbf{x}}_{1}}(\xi )={\hbar }_{1}\left( {\textbf{x}}(\xi -1+\vartheta _{1})\right) ,\\ \,^{c}\Delta _{{\textbf{a}}}^{\vartheta _{2}}{x_{2}}(\xi )={\hbar _{2}}\left( {\textbf{x}}(\xi -1+\vartheta _{2})\right) ,\,\,\,\xi =0,1,\ldots ,\\ \vdots \\ \,^{c}\Delta _{{\textbf{a}}}^{\vartheta _{{\textbf{n}}}}{x_{{\textbf{n}}}}(\xi )={\hbar }_{{\textbf{n}}}\left( {\textbf{x}}(\xi -1+\vartheta _{{\textbf{n}}})\right) , \end{array}\right. } \end{aligned}$$

*where* ${\hbar }=(\hbar _{1},\ldots ,{\hbar }_{{\textbf{n}}}):{\mathbb {R}}^{{\textbf{n}}}\mapsto {\mathbb {R}}^{{\textbf{n}}}$ *and* ${\textbf{x}}_{1}(\xi )=\left( {{\textbf{x}}_{1}}(\xi ),\ldots ,{{\textbf{x}}}_{{\textbf{n}}}(\xi )\right) ^{{\textbf{T}}}\in {\mathbb {R}}^{{\textbf{n}}}.$ *Suppose that* $\vartheta _{\iota }\in (0,1),\,\,\iota =\widetilde{1,{\textbf{n}}}$ *and* ${\mathcal {H}}$ *is the least common multiple of the denominators* ${u_{ }}_{\iota }$ *of* $\vartheta _{\iota }'s$ *having* $\vartheta _{\iota }={w_{1}}_{\iota }/{\sigma }_{\iota },\,({\sigma }_{\iota },{w_{1}}_{\iota })=1,\,{w_{1}}_{\iota },{\sigma }_{\iota }\in {\mathbb {Z}}_{+},\,\iota =1,2,\ldots ,{\textbf{n}}.$ *If every root of the equation*

11 $$\begin{aligned} det\left( diag(\wp ^{{{\mathcal {H}}}_{\vartheta _{1}}},\ldots .,\wp ^{{\mathcal {H}}_{\vartheta _{{\textbf{n}}}}})-(1-\wp ^{{\mathcal {H}}}){\mathbb {J}}\right) =0, \end{aligned}$$

*If* ${{\textbf{x}}}_{0}={{\textbf{x}}}(0)$ *occurs inside the collection* $\tilde{{\mathcal {C}}}/{\mathcal {K}}^{\xi }$, *then the straightforward solution of the structure* (9) *is locally asymptotically stable (LAS), where* $\xi =1/{\mathcal {H}},$ ${\mathbb {J}}$ *is the Jacobian matrix of* (9) *and*

12 $$\begin{aligned} {\mathcal {K}}^{\xi }=\left\{ w_{1}\in \tilde{{\mathcal {C}}}:\vert w_{1}\vert \le \left( 2\cos \frac{\vert \arg w_{1}\vert }{\xi }\right) ^{\xi }\,and\,\vert \arg w_{1}\vert \le \frac{\xi \pi }{2}\right\} . \end{aligned}$$

### Remark 3.1

The classical Hartman–Grobman linearization essentially contributes a vital part to the analysis of the locally stable characteristics of a constantly changing system’s equilibrium, which contends that the specific behaviour of an unstable system in the neighbourhood of a hyperbolic neutral state is subjectively similar to the behaviour of its linearization within the equilibrium. It is worth noting that a fractional-order equivalent for this linearization theory was discovered in^46^. If ${X_{1}}^{*}$ is a state of equilibrium of (9), i.e. $\Theta ({X_{1}})$, the linearized framework at ${X_{1}}^{*}$ is as follows:

13 $$\begin{aligned} \nabla ^{\vartheta }\Theta (X_{1})={\mathcal {J}}_{\Theta }({X_{1}}^{*})X_{1}, \end{aligned}$$

where ${\mathcal {J}}_{\Theta }({X_{1}^{*}})$ is the Jacobian matrix of the mapping $\Theta$ determined at $X_{1}^{*}$. As a result, the dynamic technique’s equilibrium ${X_{1}^{*}}$ is asymptotically stable iff the insignificant algorithm for the dynamical framework (9) is asymptotically stable^47^. Also, in view of the well-noted Matignon’s principal^47^, the dynamical fractional-order model (13) is asymptotically stable iff $\big \vert \arg (\lambda )\big \vert >\frac{\vartheta \pi }{2},$ for some eigenvalue $\lambda$ of the Jacobian matrix ${\mathcal {J}}_{\Theta }({X_{1}^{*}})$.

### Definition 3.3

(^48^) Suppose an eigenvalues of ${\mathcal {J}}_{\Theta }({X_{1}^{*}})$ fulfills $\big \vert \arg (\lambda )\big \vert >\frac{\vartheta \pi }{2}$ and some other eigenvalues holds $\big \vert \arg (\lambda )\big \vert <\frac{\vartheta \pi }{2},$ then the steady state ${X_{1}}^{*}$ is said to be a saddle node.

### Remark 3.2

According to Morris–Lecar system (3), a steady state ${X_{1}}^{*}$ is known to be a saddle of rank one if an eigenvalue of ${\mathcal {J}}_{\Theta }({X_{1}}^{*})$ is unsteady (that is $\big \vert \arg (\lambda _{1})\big \vert <\frac{\vartheta \pi }{2}$) and remaining two eigenvalues are stable $\big \vert \arg (\lambda _{2,3})\big \vert >\frac{\vartheta \pi }{2}$. However, the two eigenvalues connected to the equilibrium ${X_{1}}^{*}$ are unstable, when merely one eigenvalue stays constant, the saddle point ${X_{1}}^{*}$ is referred to as the saddle of rank two^48^.

### Qualitative evaluation of discrete fractional-order Morris–Lecar system and mathematical implications

#### Two-dimensional model

The framework (1) is an example of a two-dimensional fractional-order conductance-dependent activated structure. The discrete version of the fractional-order Morris–Lecar system can be constructed by substituting the Caputo fractional-order formulation $\,^{c}{\textbf{D}}_{{\textbf{a}}_{1}}^{{\textbf{q}}_{\iota }}$ in framework (1) with the fractional-order difference scheme defined in (6) $\,^{c}\Delta _{{\textbf{a}}_{1}}^{\Phi _{\iota }}$, which is demonstrated below:

14 $$\begin{aligned} {\left\{ \begin{array}{ll} {\mathcal {C}}\,^{c}\Delta _{{\textbf{a}}_{1}}^{\Phi _{1}}{u_{1}}(\xi )=\Im -{\bar{\Im }}({u_{1}},{u_{2}}),\\ \,^{c}\Delta _{{\textbf{a}}_{1}}^{\Phi _{2}}{u_{2}}(\xi )=\varphi \ell ({u_{1}})({u_{2}}_{\infty }({u_{1}})-{u_{2}}) \end{array}\right. } \end{aligned}$$

for $\xi \in {\mathbb {N}}_{{\textbf{a}}+1-\Phi }.$ $\Phi _{\iota },\,\iota =1,..,3$ are the fractional-order values such that $\Phi _{\iota }\in (0,1],\,\iota =1,2,3.$ Also, ${u_{1}}$ and ${u_{2}}$ are the neuron’s cell power and restricting parameter, $\Im$ is an implemented electricity, ${\bar{\Im }}({u_{1}},{u_{2}})$ is the ionized energy, $\ell ({u_{2}})$ is the frequency steady for releasing ionized pathways, and ${u_{2}}_{\infty }$ is the amount of accessible channel openings at the state of equilibrium.

The numerical procedure for the fractional discrete system can be constructed using the subsequent hypothesis:

##### Theorem 3.3

(^49^) *Suppose there is a fractional difference equation*

15 $$\begin{aligned} {\left\{ \begin{array}{ll} \,^{c}\Delta _{{\textbf{a}}}^{\Phi _{1}}\Theta (\xi )=\digamma \left( \xi +\Phi _{\iota }-1,\Theta (\xi +\Phi _{\iota }-1)\right) ,\\ \Delta ^{\eta }\Theta (\xi )={\Theta }_{\eta },\,\,\,{\textbf{q}}=\lceil \Phi _{\iota }\rceil +1, \end{array}\right. } \end{aligned}$$

*the unique solution of this initial value problem* (15) *is presented as*

16 $$\begin{aligned} \Theta (\xi )={\Theta }_{0}(\xi )+\frac{1}{\Gamma (\Phi _{\iota })}\sum \limits _{\Upsilon ={\textbf{a}}+{\textbf{q}}-\Phi _{\iota }}^{\xi -\Phi _{\iota }}(\xi -\Upsilon +1)^{(\Phi _{\iota }-1)}\digamma \left( \xi +\Phi _{\iota }-1,\Theta (\xi +\Phi _{\iota }-1)\right) ,\,\,\xi \in {\mathbb {N}}_{{\textbf{a}}+{\textbf{q}}}, \end{aligned}$$

*where*

17 $$\begin{aligned} {\digamma }_{0}(\xi )=\sum \limits _{\eta =0}^{{\textbf{q}}-1}\frac{(\xi -{\textbf{a}})^{\eta }}{\Gamma (\eta +1)}\Delta ^{\eta }\digamma ({\textbf{a}}). \end{aligned}$$

Therefore, the mathematical representation of the discrete fractional-order Morris–Lecar neural networks framework (14) is developed using the aforesaid approach:

18 $$\begin{aligned} {\left\{ \begin{array}{ll} {\mathcal {C}}{u_{1}}({\textbf{n}})={u_{1}}(0)+\frac{1}{\Gamma (\Phi _{1})}\sum \limits _{\lambda =0}^{{\textbf{n}}-1}\frac{\Gamma ({\textbf{n}}-\lambda -1+\Phi _{1})}{\Gamma ({\textbf{n}}+1-\lambda -1)}\left( \Im (\lambda )-{\bar{\Im }}({u_{1}}(\lambda ),{u_{2}}(\lambda ))\right) ,\\ {u_{2}}({\textbf{n}})={u_{2}}(0)+\frac{1}{\Gamma (\Phi _{2})}\sum \limits _{\lambda =0}^{{\textbf{n}}-1}\frac{\Gamma ({\textbf{n}}-\lambda -1+\Phi _{2})}{\Gamma ({\textbf{n}}+1-\lambda -1)}\left( \varphi \ell ({u_{1}}(\lambda ))\left( {u_{2}}_{\infty }({u_{1}}(\lambda ))-{u_{2}}(\lambda )\right) \right) , \end{array}\right. } \end{aligned}$$

where ${u_{1}}(0)$ and ${u_{2}}(0)$ indicate initial conditions. This is an entirely novel kind of Morris–Lecar system that has “memory impacts.” As shown in (18), the configurations of ${u_{1}}({\textbf{n}})$ and ${u_{2}}({\textbf{n}})$ are interdependent concerning every previous factor ${u_{1}}(0),\,{u_{1}}(1),\ldots ,{u_{1}}({\textbf{n}}-1)$ and ${u_{2}}(0),\,{u_{2}}(1),\ldots ,{u_{2}}({\textbf{n}}-1).$

#### Three-dimensional model

The framework (3) is an example of a three-dimensional fractional-order conductance-dependent activated structure. The discrete version of the fractional-order Morris–Lecar system can be constructed by substituting the Caputo fractional-order formulation $\,^{c}{\textbf{D}}_{{\textbf{a}}_{1}}^{{\textbf{q}}_{\iota }}$ in framework (3) with the fractional-order difference scheme defined in (6) $\,^{c}\Delta _{{\textbf{a}}_{1}}^{\Phi _{\iota }}$, which is demonstrated below:

19 $$\begin{aligned} {\left\{ \begin{array}{ll} {\mathcal {C}}\,^{c}\Delta _{{\textbf{a}}_{1}}^{\Phi _{1}}{u_{1}}(\xi )=\Im ({u_{3}})-{\bar{\Im }}({u_{1}},{u_{2}}),\\ \,^{c}\Delta _{{\textbf{a}}_{1}}^{\Phi _{2}}{u_{2}}(\xi )=\varphi \ell ({u_{1}},{u_{3}})(\bar{{u_{2}}}({u_{1}},{u_{3}})-{u_{2}}),\\ \,^{c}\Delta _{{\textbf{a}}_{1}}^{\Phi _{3}}{u_{3}}(\xi )=\theta ({u_{1}}+{\mathcal {F}}_{0}) \end{array}\right. } \end{aligned}$$

for $\xi \in {\mathbb {N}}_{{\textbf{a}}+1-\Phi }.$ $\Phi _{\iota },\,\iota =1,..,3$ are the fractional-order values such that $\Phi _{\iota }\in (0,1],\,\iota =1,2,3.$

## Dynamic analysis of Morris–Lecar model

In this section, we will investigate whether the formerly suggested discrete fractional-order Morris–Lecar neural network model (19) is steady or in chaos in both instances: commensurate and non-commensurate fractional-orders, respectively. The present research will make use of an assortment of computational techniques, among them bifurcation illustrations and the visual representation of phase profiles in multifaceted estimations. In addition, we use the reduced order evaluation to determine whether or not chaos is present.

### Commensurate fractional-order for two-dimensional Morris–Lecar model

In what follows, the robustness of the stable state points within the discrete fractional-order Morris–Lecar neural networks model (18) with commensurate fractional-order is investigated. For the purpose of determining the system’s steady state, we consider the model (18):

20 $$\begin{aligned} {\bar{\Im }}({u_{1}},{u_{2}})={\mathcal {W}}_{Ca}{{\textbf{r}}}_{\infty }({u_{1}})({u_{1}}-1)+{\mathcal {W}}_{{\mathcal {K}}}{u_{2}}({u_{1}}-{\mathcal {F}}_{k_{1}})+{\mathcal {W}}_{{\mathcal {L}}}({u_{1}}-{\mathcal {F}}_{{\mathcal {L}}}), \end{aligned}$$

and $${{\textbf{r}}}_{\infty }({u_{1}})=\frac{1}{2}\left( 1+\tanh \left( \frac{{u_{1}}-{\mathcal {F}}_{1}}{{\mathcal {F}}_{2}}\right) \right) ,\,\,{u_{2}}_{\infty }({u_{1}})=\frac{1}{2}\left( 1+\tanh \left( \frac{{u_{1}}-{\mathcal {F}}_{3}}{{\mathcal {F}}_{4}}\right) \right) ,\,\ell ({u_{1}})=\cosh \left( \frac{{u_{1}}-{\mathcal {F}}_{3}}{2{\mathcal {F}}_{4}}\right) .$$

The mathematical method’s solutions are the fixed points of model (18):

$$\begin{aligned} \Im ={\bar{\Im }}({u_{1}},{u_{2}}),\,\,{u_{2}}={u_{2}}_{\infty }({u_{1}}), \end{aligned}$$

equivalently,

$$\begin{aligned} \Im ={\bar{\Im }}({u_{1}},{u_{2}}_{\infty }):=\Im _{\infty }({u_{1}}),\,\,{u_{2}}={u_{2}}_{\infty }({u_{1}}). \end{aligned}$$

The mapping $\Im ({u_{1}})$ has a handful of fundamental characteristics:

- $${\textbf{A}}_{1}:\,\Im _{\infty }\in {\mathcal {C}}^{1}({\mathbb {R}});$$
- $${\textbf{A}}_{2}:\,\lim \limits _{{u_{1}}\mapsto -\infty }\Im _{\infty }({u_{1}})=-\infty \,\,\,and\,\,\,\lim \limits _{{u_{1}}\mapsto \infty }\Im _{\infty }({u_{1}})=\infty ;$$
- $${\textbf{A}}_{3}:\,\Im _{\infty }\,has\,only\,two\,real\,roots\,{u_{1}}_{\max }<{u_{1}}_{\min }.$$

It is critical to emphasize that these features are fulfilled in the specific scenario of the Morris–Lecar neural networks that includes the mapping $\Im ({u_{1}},{u_{2}})$ supplied by (18).

We indicate $\Im _{\max }=\Im _{\infty }$ as ${u_{1}}_{\max }$, and $\Im _{\min }=\Im _{\infty }$ as ${u_{1}}_{\min }$. Then $\Im$ increases on the ranges $(-\infty ,{u_{1}}_{\max }]$ and $[{u_{1}}_{\min },\infty )$ and decreases on the time frame $({u_{1}}_{\max },{u_{1}}_{\min }).$

As a result, subject to the exterior input $\Im$, there exist three separate sets of equilibrium points, signified by $({u_{1}}_{\iota }(\Im ),{u_{2}}_{\infty }({u_{1}}_{\iota }(\Im ))),\,\iota \in \{1,2,3\},$ which included:

$$\begin{aligned}{} & {} \Im _{1}=\Im _{\infty }\vert _{(-\infty ,{u_{1}}_{\max }]},\,\,{u_{1}}_{1}:(-\infty ,\Im _{\max }]\mapsto (-\infty ,{u_{1}}_{\max }],\,\,{u_{1}}_{1}(\Im )=\Im _{1}^{-1}(\Im ),\\{} & {} \Im _{2}=\Im _{\infty }\vert _{({u_{1}}_{\min },{u_{1}}_{\max })},\,\,{u_{1}}_{2}:(\Im _{\min },\Im _{\max })\mapsto ({u_{1}}_{\min },{u_{1}}_{\max }),\,\,{u_{1}}_{2}(\Im )=\Im _{2}^{-1}(\Im ),\\{} & {} \Im _{3}=\Im _{\infty }\vert _{[{u_{1}}_{\min },\infty )},\,\,{u_{1}}_{3}:[\Im _{\min },\infty )\mapsto ({u_{1}}_{\min },\infty ),\,\,{u_{1}}_{3}(\Im )=\Im _{3}^{-1}(\Im ). \end{aligned}$$

#### Remark 4.1

As an outcome, any of the scenarios listed below are possible:

- When $\Im <\Im _{\min }$ or $\Im >\Im _{\max },$ then model (18) has only one steady state.
- When $\Im =\Im _{\min }$ or $\Im =\Im _{\max },$ then model (18) has two one steady states.
- When $\Im \in (\Im _{\min },\Im _{\max }),$ then model (18) has three steady states.

#### Stability of steady states

For an undetermined equilibrium state $({u_{1}}^{*},{u_{2}}^{*})=({u_{1}}^{*},{u_{2}}_{\infty }({u_{1}}^{*})),$ the Jacobian matrix corresponding to the dynamics (18) is:

$$\begin{aligned} {\textbf{J}}=\begin{pmatrix} -{\bar{\Im }}_{{u_{1}}}\left( {u_{1}}^{*},{u_{2}}_{\infty }({u_{1}}^{*})\right) /{\mathcal {C}}&{}-{\bar{\Im }}_{{u_{2}}}\left( {u_{1}}^{*},{u_{2}}_{\infty }({u_{1}}^{*})\right) /{\mathcal {C}}\\ \varphi \ell ({u_{1}}^{*}){u_{2}}_{\infty }^{\prime }({u_{1}}^{*})&{}-\varphi \ell ({u_{1}}^{*}) \end{pmatrix}. \end{aligned}$$

In the present situation, the essentials that are required for a steady state’s asymptotic stability $({u_{1}}^{*},{u_{2}}^{*})$ minimize to the subsequent variants^50^:

$$\begin{aligned} \zeta ({u_{1}}^{*})>0\,\,\,and\,\,\chi ({u_{1}}^{*})<2\sqrt{\zeta ({u_{1}}^{*})}\cos \left( \frac{\vartheta \pi }{2}\right) , \end{aligned}$$

where

$$\begin{aligned}{} & {} \chi ({u_{1}}^{*})=trac({\textbf{J}})=-\frac{1}{{\mathcal {C}}}{\bar{\Im }}_{{u_{1}}}({u_{1}}^{*},{u_{2}}_{\infty }({u_{1}}^{*}))-\varphi \ell ({u_{1}}^{*}),\\{} & {} \zeta ({u_{1}}^{*})=\det ({\textbf{J}})=\frac{\varphi }{{\mathcal {C}}}\ell ({u_{1}}^{*})\left[ {\bar{\Im }}_{{u_{1}}}\left( {u_{1}}^{*},{u_{2}}_{\infty }({u_{1}}^{*})\right) +{u_{2}}_{\infty }^{\prime }({u_{1}}^{*}){\bar{\Im }}_{{u_{2}}}\left( {u_{1}}^{*},{u_{2}}_{\infty }({u_{1}}^{*})\right) \right] =\frac{\varphi }{{\mathcal {C}}}\ell ({u_{1}}^{*})\Im _{\infty }^{\prime }({u_{1}}^{*}). \end{aligned}$$

We commence by pointing out that the following branch of equilibria is entirely unsteady. In fact, any state of equilibrium $\left( {u_{1}}_{2}(\Im ),{u_{2}}_{\infty }({u_{1}}_{2}(\Im ))\right)$ alongside $\Im \in (\Im _{\min },\Im _{\max })$ adheres to $\Im ^{\prime }({u_{1}}_{2}(\Im ))<0,$ thereby achieving $\zeta ({u_{1}}_{2}(\Im ))<0.$ Furthermore, irrespective of what fractional-order is taken into account in structure (18), the minus sign of the aforesaid system ensures that every steady state of the subsequent process is the saddle node.

On the contrary, the determinant of the Jacobian is straightforward to determine to be non-negative for both the initial and third segments of steady states. As a result, the trace $\chi$ has a strong influence on the sturdiness of the steady states. If $\chi ({u_{1}}^{*})<0$, the steady state $({u_{1}}^{*},{u_{2}}^{*})$ is evidently asynchronously stable, regardless of the fractional-order $\vartheta$ examined in framework (18). Whenever, $\chi ({u_{1}}^{*})\ge 0$, a steady state $({u_{1}}^{*},{u_{2}}^{*})$ of the initial or third branch is asynchronously stable, iff

21 $$\begin{aligned} \vartheta <\vartheta ^{*}({u_{1}}^{*})=\frac{2}{\pi }\cos ^{-1}\left( \frac{\chi ({u_{1}}^{*})}{2\sqrt{\zeta ({u_{1}}^{*})}}\right) . \end{aligned}$$

Furthermore, we will make the assumption that ${\mathcal {F}}_{k_{1}}<{u_{1}}_{\max }<{u_{1}}_{\min }<1.$ We effortlessly assess:

$$\begin{aligned} {\bar{\Im }}_{{u_{1}}}({u_{1}},{u_{2}}_{\infty }({u_{1}}))={\mathcal {W}}_{Ca}\left[ {{\textbf{r}}}_{\infty }^{\prime }({u_{1}})({u_{1}}-1)+{{\textbf{r}}}_{\infty }({u_{1}})\right] +{\mathcal {W}}_{k_{1}}{u_{2}}_{\infty }({u_{1}})+{\mathcal {W}}_{{\mathcal {L}}}, \end{aligned}$$

and so that, if $({u_{1}}^{*},{u_{2}}^{*})=\left( {u_{1}}^{*},{u_{2}}_{\infty }({u_{1}}^{*})\right)$ is a steady state of the third branch such that ${u_{1}}^{*}>1,$ as a result of this ${\bar{\Im }}_{{u_{1}}}\left( {u_{1}}^{*},{u_{2}}_{\infty }({u_{1}}^{*})\right) >0$ and therefore $\chi ({u_{1}}^{*})<0.$

Additionally, we are able to communicate as

$$\begin{aligned} {\bar{\Im }}_{{u_{1}}}\left( {u_{1}},{u_{2}}_{\infty }({u_{1}})\right) =\Im _{\infty }^{\prime }({u_{1}})-{u_{2}}_{\infty }^{\prime }({u_{1}}){\bar{\Im }}_{{u_{2}}}\left( {u_{1}},{u_{2}}_{\infty }({u_{1}})\right) =\Im _{\infty }^{\prime }({u_{1}})-{u_{2}}_{\infty }^{\prime }({u_{2}}){\mathcal {W}}_{{\mathcal {K}}}({u_{1}}-{\mathcal {F}}_{{\mathcal {K}}}), \end{aligned}$$

and thus, if $({u_{1}}^{*},{u_{2}}^{*})$ is a steady state of the initial branch, which indicates ${u_{1}}^{*}<{\mathcal {F}}_{{\mathcal {K}}}.$ It is concluded that ${\bar{\Im }}_{{u_{1}}}\left( {u_{1}}^{*},{u_{2}}_{\infty }({u_{1}}^{*})\right) >0$ and repeating the same argument, we attain $\chi ({u_{1}}^{*})<0.$

In accordance with the aforementioned computation, we further note that:

$$\begin{aligned} \chi ({u_{1}}_{{\textbf{r}}})=-\frac{1}{{\mathcal {C}}}\left[ \Im _{\infty }^{\prime }({u_{1}}_{\infty })-{u_{2}}_{\infty }^{\prime }({u_{1}}_{{\textbf{r}}}){\mathcal {W}}_{{\mathcal {K}}}({u_{1}}_{{\textbf{r}}}-{\mathcal {F}}_{{\mathcal {K}}})\right] -\varphi \ell ({u_{1}}_{{\textbf{r}}})=\frac{1}{{\mathcal {C}}}\left[ {u_{2}}_{\infty }^{\prime }({u_{1}}_{{\textbf{r}}}){\mathcal {W}}_{{\mathcal {K}}}({u_{1}}_{{\textbf{r}}}-{\mathcal {F}}_{{\mathcal {K}}})\right] -\varphi \ell ({u_{1}}_{{\textbf{r}}}), \end{aligned}$$

such as ${u_{1}}_{{\textbf{r}}}={u_{1}}_{\max }$ or ${u_{1}}_{{\textbf{r}}}={u_{1}}_{\min },$ and considering that is sufficiently small, the variant $\chi ({u_{1}}_{{\textbf{r}}})>0$ is capable of being noticed. As a result, the mapping $\chi ({u_{1}})$ may have two roots, ${u_{1}}^{\prime }\in ({\mathcal {F}}_{{\mathcal {K}}},{u_{1}}_{\max })$ and ${u_{1}}^{\prime \prime }\in ({u_{1}}_{\min },1),$ respectively. In view of the numerical information, we might additionally suppose that when such roots occur, they constitute something particular within the preceding time frames.

Finally, the stability of equilibria conditions is only affected by fractional-order in the subsequent three scenarios:

(${\textbf{c}}_{1}$) the steady state is associated with the initial region and ${u_{1}}^{*}\in ({u_{1}}^{\prime },{u_{1}}_{\max });$

(${\textbf{c}}_{2}$) the steady state is associated with the third region and ${u_{1}}^{*}\in ({u_{1}}_{\min },{u_{1}}^{\prime \prime });$

(${\textbf{c}}_{3}$) the steady state is associated with the second region is unstable regardless of fractional-order.

Now, the threshold $\vartheta ^{*}$ provided by (21) is equivalent to a bifurcation in the present instance (that is., the Jacobian matrix possesses two sets of imaginary type eigenvalues that result in $\big \vert \arg (\lambda )\big \vert =\frac{\vartheta \pi }{2}$). In a nutshell, contingent on the fractional-order evaluated in framework (18), the states of the Hopf bifurcation are indicated in the $(\Im ,{u_{1}})$-plane, located on the primary and/or third branches, accordingly. Evidently, such will have an extremely significant influence on the form of spiked and brimming behaviour in the two-dimensional framework (18) and the three-dimensional slow-fast framework, as will be revealed in the subsection that follows.

**Figure 1:**
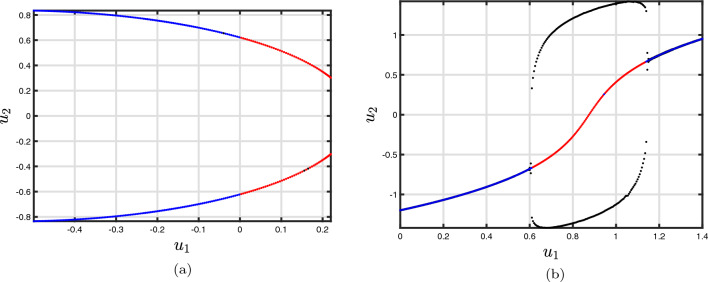
According to group **i** and **ii**, bifurcation cases of the two-dimensional Morris–Lecar neural networks. (**a**) $\Im$ as a bifurcation factor: ($\Im =90,\,\vartheta =0.97$) and ($\Im =40,\,\vartheta =0.1$) indicate the presence of Hopf and saddle component type plots in the framework (18), respectively. The blue and red lines represent the structure’s balance and imbalance branches, respectively. (**b**) reflects the appearance of an unsteady limit process.

**Figure 2:**
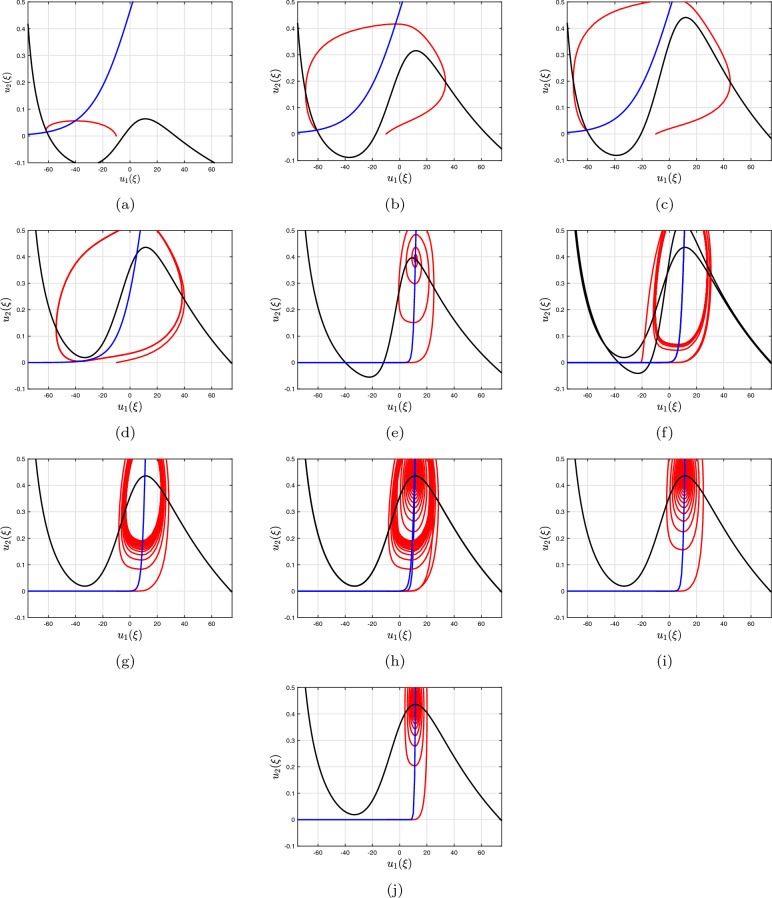
Phase depictions (which incorporates nullclines) in the $({u_{1}},{u_{2}})$-plane for the two-dimensional Morris–Lecar neural networks system having discrete fractional-order commensurate technique: (**a**) $\vartheta =1$, (**b**) $\vartheta =0.97$, (**c**) $\vartheta =0.92$, (**d**) $\vartheta =0.89$, (**e**) $\vartheta =0.85$, (**f**) $\vartheta =0.82$, (**g**) $\vartheta =0.79$, (**h**) $\vartheta =0.75$, (**i**) $\vartheta =0.72$, (**j**) $\vartheta =0.70.$.

**Figure 3:**
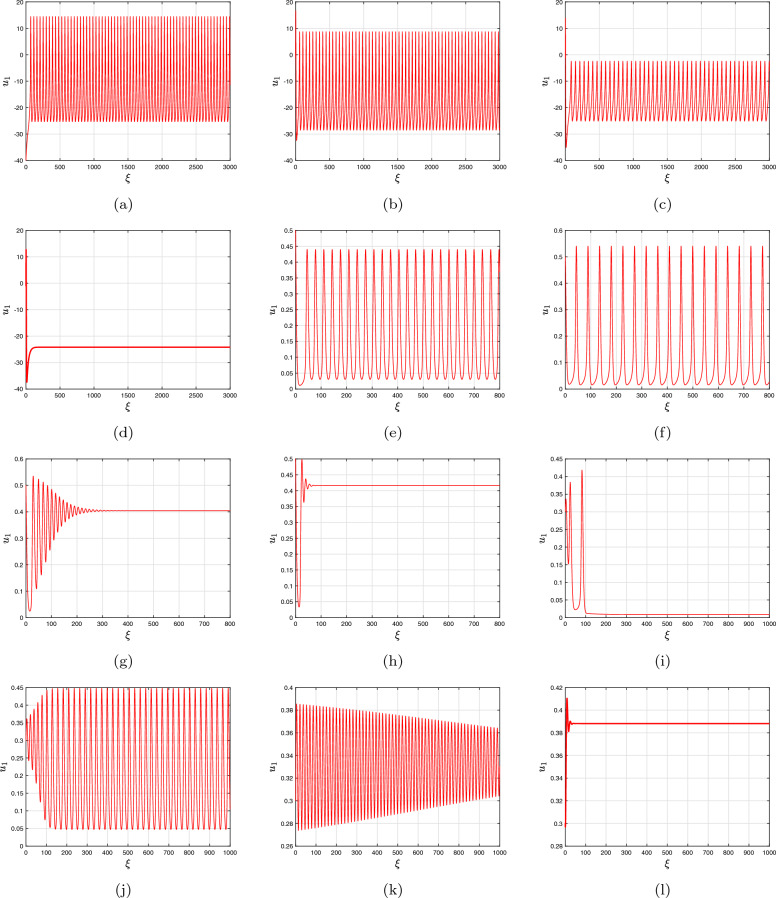
Time analysis of group **i** and **ii** of discrete fractional-order Morris–Lecar neural networks (18) for various fractional-orders (**a**-**d**) $\vartheta =1,\,0.97,\,0.95,\,0.93,\,0.90$ having $\Im =43;$ (**e**–**h**) $\vartheta =1,\,0.97,\,0.95,\,0.93,\,0.90$ having $\Im =50$ including the specification of group **i** and **ii**; (**i**–**l**) $\vartheta =1,\,0.97,\,0.95,\,0.93,\,0.90$ having $\Im =90$ including the specification of group **iii**.

As an illustration, take into account $\Im$ as a bifurcation variable while looking at the bifurcation setting of the conventional two-dimensional Morris–Lecar neural networks framework that includes Hopf-bifurcating highlights (Fig. 1a). Consequently, we examined the influence of fractional-order on the behaviour of the framework (18) and demonstrated the way that it stabilizes the entire structure as the quantity decreases (Fig. 1b). The period depictions of the two-dimensional Morris–Lecar structure using its settings for Group **ii** are then shown in Fig. 2a–j for various fractional-order outcomes. In the present scenario, the framework has exclusively one unsteady equilibrium point, $({u_{1}}^{*},{u_{2}}^{*})=(6.23101, 1/24,232)$ (at the point of convergence of the nullclines), which is positioned at the subsequent location. According to the procedure (21), the significant worth of the fractional-order that describes the Hopf-bifurcation at the steady state is $\vartheta ^{*}=0.89342.$ A large-amplitude constraint process attractor, similar to spiked behaviour, can be observed in the classical situation, $\vartheta =1$. The large-amplitude appealing quasiperiodic restrict process addresses the unsteady state of equilibrium as the fractional-order reduces, and as it tackles the important threshold for Hopf splitting, an additional quasiperiodic pass over arises, featuring smaller-amplitude fluctuations near the point of stability in addition to large-amplitude rises. When $\vartheta <\vartheta ^{*},$ the balance of power turns into asynchronously steady. Furthermore, we demonstrate associated data sets to additionally validate the mathematical findings, mentioning that the fractional-order determined thresholds are $\vartheta ^{*}=0.83241$ for group **i** and $\vartheta ^{*}=0.96720$ for group **ii** presented in Fig. 3a–d for $\Im =43,$ Fig. 3e–h for $\Im =50$ and Fig. 3i–l for $\Im =90$ with the settings of Group **iii** for various fractional-order outcomes, respectively.

### Commensurate fractional-order for three-dimensional Morris–Lecar model

In accordance with the already mentioned features, the three-dimensional slow-fast fractional-order designs (19) are capable of being composed in (20) with the parameter settings ${\bar{\Im }}({u_{1}},{u_{2}})$ as follows:

22 $$\begin{aligned} \bar{{u_{2}}}({u_{1}},{u_{3}})=\frac{1}{2}\left( 1+\tanh \left( \frac{{u_{1}}-{\mathcal {F}}_{3}({u_{3}})}{{\mathcal {F}}_{4}}\right) \right) ,\,\,\,\,\,\,{\bar{\ell }}({u_{1}},{u_{3}})=\cosh \left( \frac{{u_{1}}-{\mathcal {F}}_{3}({u_{3}})}{2{\mathcal {F}}_{4}}\right) . \end{aligned}$$

We are taking on the fact that $\Im ({u_{3}})$ and ${\mathcal {F}}_{3}({u_{3}})$ are lowering functions and that ${\mathcal {F}}_{0}+{\mathcal {F}}_{{\mathcal {K}}}<0$ (based on the variable distinction assumed). $({u_{1}}^{*},{u_{2}}^{*},{u_{3}}^{*})$ is the particular steady states of framework (19), where ${u_{1}}^{*}=-{\mathcal {F}}_{0},\,{u_{2}}^{*}=\bar{{u_{2}}}({u_{1}}^{*},{u_{3}}^{*})$ and ${u_{3}}^{*}$ is the distinctive root of the completely non-increasing mapping ${u_{3}}\mapsto \Im ({u_{3}})-{\bar{\Im }}(-{\mathcal {F}}_{0},\bar{{u_{2}}}(-{\mathcal {F}}_{0},{u_{3}})).$

At $({u_{1}}^{*},{u_{2}}^{*},{u_{3}}^{*}),$ the Jacobian matrix for system (19) is defined as

23 $$\begin{aligned} {\textbf{J}}=\begin{pmatrix} -{\bar{\Im }}_{{u_{1}}}({u_{1}}^{*},{u_{2}}^{*})/{\mathcal {C}}&{}-{\bar{\Im }}_{{u_{2}}}({u_{1}}^{*},{u_{2}}^{*})/{\mathcal {C}}&{}{\Im ^{\prime }}({u_{3}}^{*}/{\mathcal {C}}\\ \varphi {\bar{\ell }}({u_{1}}^{*},{u_{3}}^{*})\bar{{u_{2}}}_{{u_{1}}}({u_{1}}^{*},{u_{3}}^{*})&{}-\varphi {\bar{\ell }}({u_{1}}^{*},{u_{3}}^{*})&{}\varphi {\bar{\ell }}({u_{1}}^{*},{u_{3}}^{*})\bar{{u_{2}}}_{{u_{3}}}({u_{1}}^{*},{u_{3}}^{*})\\ {u_{1}}&{}0&{}0 \end{pmatrix} \end{aligned}$$

and the characteristic polynomial of the aforesaid system (23) is:

24 $$\begin{aligned} \lambda ^{3}+\varpi _{1}\lambda ^{2}+\varpi _{2}\lambda +\varpi _{3}=0, \end{aligned}$$

where

$$\begin{aligned} \varpi _{1}= & {} \frac{1}{{\mathcal {C}}}{\bar{\Im }}_{{u_{1}}}({u_{1}}^{*},{u_{2}}^{*})+\varphi {\bar{\ell }}({u_{1}}^{*},{u_{3}}^{*}),\nonumber \\ \varpi _{2}{} & {} =\frac{\varphi }{{\mathcal {C}}}{\bar{\ell }}({u_{1}}^{*},{u_{3}}^{*})\left[ {\bar{\Im }}_{{u_{1}}}({u_{1}}^{*},{u_{2}}^{*})+{\bar{\Im }}_{{u_{2}}}({u_{1}}^{*},{u_{2}}^{*})\bar{{u_{2}}}_{{u_{1}}}({u_{1}}^{*},{u_{3}}^{*})\right] -\frac{{u_{1}}}{{\mathcal {C}}},\Im ^{\prime }({u_{3}}^{*}),\\ \varpi _{3}= & {} \frac{{u_{1}}\varphi }{{\mathcal {C}}}{\bar{\ell }}({u_{1}}^{*},{u_{3}}^{*})+\varphi {\bar{\ell }}({u_{1}}^{*},{u_{3}}^{*})\left[ {\bar{\Im }}_{{u_{2}}}({u_{1}}^{*},{u_{2}}^{*})+\bar{{u_{2}}}({u_{1}}^{*},{u_{3}}^{*})-\Im ^{\prime }({u_{3}}^{*})\right] >0. \end{aligned}$$

The non-negativity of the factor $\varpi _{3}$ as a result of

$$\begin{aligned} {\bar{\Im }}_{{u_{2}}}({u_{1}}^{*},{u_{2}}^{ast})={\mathcal {W}}_{{\mathcal {K}}}({u_{1}}^{*}-{\mathcal {F}}_{{\mathcal {K}}})=-{\mathcal {W}}_{{\mathcal {K}}}({\mathcal {F}}_{0}+{\mathcal {F}}_{{\mathcal {K}}})>0. \end{aligned}$$

Because $\varpi _{3}>0,$ the product of the aforesaid system’s eigenvalues is non-positive, the first of the eigenvalues is a non-positive actual quantity, while the remaining two can be intricate conjugate or actual and possess the same symbolically. We are additionally assuming that no fewer than a single of the factors $\varpi _{1}$ or $\varpi _{2}$ is non-positive (depending on the setting established according to supposition), indicating that the Routh–Hurwitz principle for the specific polynomial is not fulfilled. As a result, we differentiate two scenarios using the ${\textbf{D}}$ discriminant property of the (24):

- When ${\textbf{D}}>0,$ the Jacobian matrix ${\textbf{J}}$ has only a non-positive and two non-negative eigenvalues and the steady state $({u_{1}}^{*},{u_{2}}^{*},{u_{3}}^{*})$ is a saddle node of measure two regardless of fractional-order (for example, in the situation of group **i** and **ii** attributes).
- When ${\textbf{D}}<0$, the Jacobian matrix ${\textbf{J}}$ possesses a single adverse eigenvalue and two multifaceted conjugate eigenvalues that have favourable actual elements (for example, in the instance of group **iii** attributes). As a result, there exists an essential fractional-order $\vartheta ^{*}$ appreciation, which means the steady state $({u_{1}}^{*},{u_{2}}^{*},{u_{3}}^{*})$ is asynchronously steady $\vartheta <\vartheta ^{*}$ regardless of and unpredictable for $\vartheta ^{*}>\vartheta$. A Hopf-type bifurcation happens in the vicinity of the steady state at $\vartheta =\vartheta ^{*},$ leading to the emergence of relentless fluctuations. The critical threshold $\vartheta ^{*}$ is determined employing the approach described in ($\vartheta ^{*}= 0.7391$ for group **iii**).

### Dynamics of various oscillating reactions

We begin with fractional-order group **i** and **ii** single Morris–Lecar neural networks and subsequently proceed on to slow-quick behaviour. The sudden increases have been generated using an individual framework, and the cell membrane power interactions are dependent upon the power-controlled conductances. The feedback signal is referred to as **i**. To identify group **i** and **ii** capabilities, we adjusted the fractional-order value, utilizing various setting regimes: a stimulating spiked region and a rapid spiked region. We then demonstrate the variations of the power operations over an extensive magnitude as well as the sudden increase in regularity and responsive consequences. We investigated two distinct and appropriate energy stimuli: $\Im =42$ and $\Im =50$ for group **i** neurons and $\Im =90$ for group **ii** neurons. We settled on these sorts of stimulus objects because they exhibit active spikes as well as quick increases for integer-order impacts; nevertheless, whenever we modify the situation in the fractional domain, the evolving system generates distinctions within the emitted capabilities that we have not previously examined. The bifurcating assessment is carried out, the computational outcomes are reinforced by the robustness research, and the thoughtful and computational outcomes are well acknowledged.

**Figure 4:**
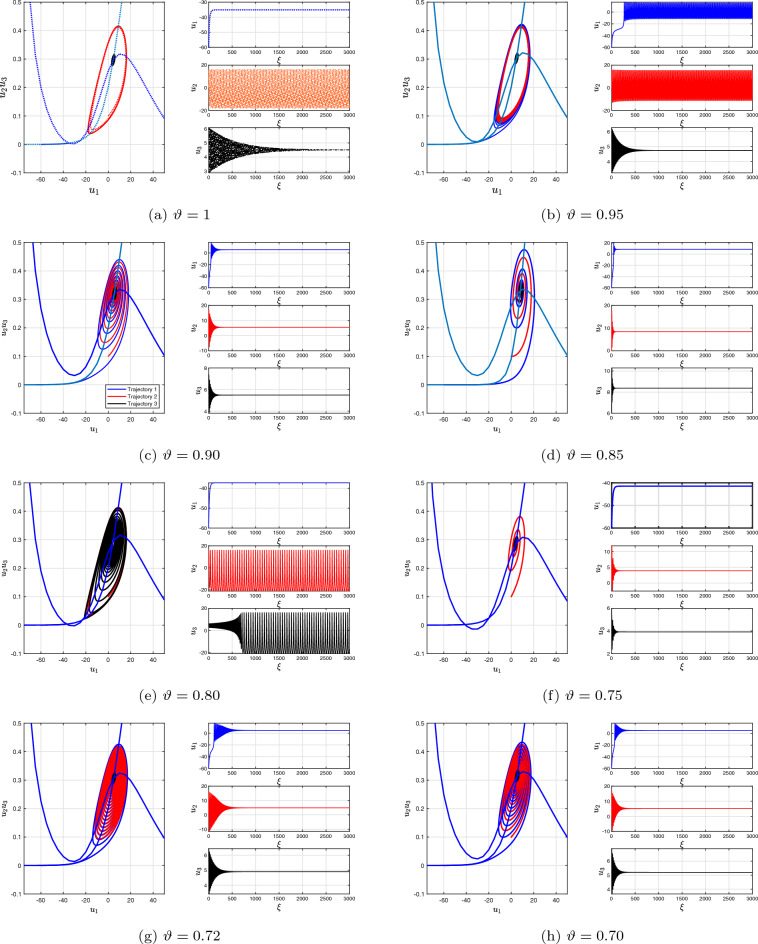
Bifurcation plot as a single panel and time dependent plots of slow-swift active three-dimensional discrete fractional-order Morris–Lecar neural network model (19) for various fractional-orders, $\vartheta =1,\,0.95,\,0.90,\,0.85,\,0.80,\,0.75,\,0.72,\,0.70$ with model specifications group **i**, **ii** and **iii**.

Low-frequency surges are unable to be produced by categorized **ii**-activated neural networks. The neurons are a combination of dormant or ablaze with a stream of surges along with a greater rate in response to resilient feedback electricity. Given $\vartheta =1$ for $\Im =90,$ only one Morris–Lecar neural network via attribute category **ii** exhibits swift spikes (see Fig. 4a). It produces mutated monarch butterfly optimizations and mixed mode oscillations when values of $\vartheta =0.95$ and 0.90 are decreased (see Fig. 4b and c, respectively. In addition to another reduction of $\vartheta =0.85,$ the explosions turn to conventional mixed mode oscillations, but they depict mixed mode oscillations that have a reduced terminating speed, that is, the period between surge time frame boosts (see Fig. 4d). The framework subsequently enters an inactive state with a value of $\vartheta =0.80$ and merges to its determined location (see Fig. 4e).

Therefore, we examine the activated Morris–Lecar neural network cells of group **i** via variable groups **i** and **ii**. Whenever excited, the integer-order neuronal cells exhibits tonic spiking; while the supplied challenge energy is on ($\Im =42$), the nerve cell persists, displaying a sequence of surges known as dominant spikes. Following this, as the fractional-order reduces to $\vartheta =0.75$, it exhibits dominant spikes (see Fig. 4f), but the inter-spike time frame goes up for $u_{1},\,u_{2}$ and $u_{3}$ (see Fig. 4f), implying that the activation rate declines. Additionally, drops of $\vartheta =0.75$ and 0.72 produce conventional overflowing (see Fig. 4g), followed by consistently overflowing that has a reduced terminating rate. After that, it enters a state of inertia alongside a lower fractional-order $\vartheta =0.70$, which agrees well with the experimental findings (see Fig. 4h). The classical solitary neuronal cells then exhibit rapidly spikes via setting group **ii** whereas the supplied stimulation is on $\Im =42.$ considering a reduction of $\vartheta =0.78$, the explosions transform into conventional bursting; nevertheless, with $\vartheta = 0.80$ and 0.76, the number of explosions declines while a greater surge is produced. Ultimately, at $\vartheta =0.71$, it enters a dormant phase.

We presently expand our investigation via the activated slow-fast independent three-dimensional Morris–Lecar neural network cell simulation (19) in the fractional context to examine various setting structures that provide distinctive overflowing characteristics, that is, the quantity of surges throughout every explosion fluctuates via distinct-sized intensity levels. Given setting **i**, an individual Morris–Lecar simulation creates overflowing that has many surges for every explosion at $\vartheta =1,$ but in diminishes of $\vartheta =0.95$ and 0.85, the explosion regularity declines in an extended time frame, i.e., the inter-spike time frame improves during each explosion and intensity for every rise lowers in tandem bursts (see the bifurcation plots of Fig. 4a,b,d). The distinctive point of reference for the aforementioned parameter setting is a saddle corresponding to measure two. Spike’s condition rate modification has been noticed alongside decreasing fractional-order values. Subsequently, following a greater reduction of $\vartheta =0.72$, it induces an additional surge in the rate of adjustment (see the bifurcation plots on the left single panel in Fig. 4g). Furthermore, the standard independent neuronal cell simulation exhibits overflow via setting **ii**. Through reducing $\vartheta =0.90$ and 0.80, it exhibits different periods of overflowing and spikes in behaviour via rise regularity modification and initially surge delays at $\vartheta = 0.70$ (see the bifurcation plots on the left single panel in Fig. 4c,e,h). Ultimately, within group **iii**, an individual neuronal cell switches from overflowing to swiftly spiked while changing via one to $\vartheta = 0.95$ and 0.85. With $\vartheta = 0.72$, it enters an uninterrupted state of equilibrium, that is, it settles at the technique’s locally asynchronous centre of gravity (see Fig. 4g).

### Incommensurate discrete fractional-order two-dimensional Morris–Lecar neural networks model

**Figure 5:**
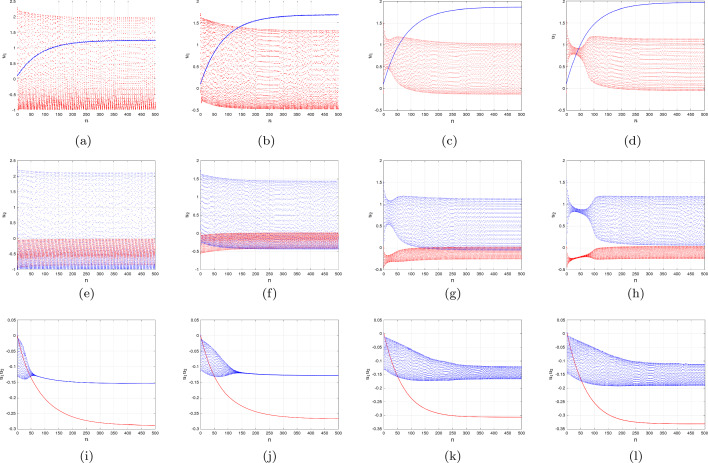
Time-analysis plots interactions of an independently associated ensemble of group **i** and **ii** discrete fractional-order two-dimensional Morris–Lecar neural network (18) with various fractional-orders; (**a**-**d**) Group **i**: $\vartheta _{1}=\ldots =\vartheta _{65}=1$ and $\Phi _{66}=\ldots =\Phi _{100}=0.80$ having ${\mathcal {W}}_{\epsilon }=0.0005,\,0.005,\,0.05,\,1.$ (**e**–**h**) Group **ii**: $\vartheta _{1}=\ldots =\vartheta _{65}=0.95$ and $\Phi _{66}=\ldots =\Phi _{100}=0.80$ having ${\mathcal {W}}_{\epsilon }=0.0005,\,0.007,\,0.05,\,1.$ (**i**-**l**) Group **iii**: $\vartheta _{1}=\ldots =\vartheta _{65}=0.84$ and $\Phi _{66}=\ldots =\Phi _{100}=0.72$ having ${\mathcal {W}}_{\epsilon }=0.0005,\,0.001,\,0.05,\,1.$ We selected these two structures from two samples to display the time indications. The interval for the assessment of the particular components identified via a blue region is selected from a subclass of components in inactive states (when ${\mathcal {W}}_{\epsilon }=0$). The red indicator is drawn from points that were maintained in jumping assertions in the dearth of interaction.

Here, we examine the evolution of a discrete fractional-order Morris–Lecar neural network simulation using an unpredictable network configuration, whereby every neural network communicates unintentionally and is assigned an interaction likelihood ${\tilde{p}}$. To identify computational modelling, we build an Erdös-Rényi network^51^ of ${\textbf{N}}=100$ Morris–Lecar oscillating components with an average node-index of $\langle \eta \rangle \approx 7$. A comprehensive review of the structure design will be provided in the sections that follow:

26 $$\begin{aligned} {\left\{ \begin{array}{ll} {\mathcal {C}}\,^{c}\Delta _{{\textbf{a}}}^{\vartheta _{\iota }}{u_{1}}(\xi )=-1/2{\mathcal {W}}_{Ca}({u_{1}}_{\iota }-{\mathcal {F}}_{Ca})\left( (1+\tanh ({u_{1}}_{\iota }-{\mathcal {F}}_{1}))/{\mathcal {F}}_{2}\right) -{u_{2}}_{\iota } {\mathcal {W}}_{{\mathcal {K}}}({u_{1}}_{\iota }-{\mathcal {F}}_{{\mathcal {K}}})\\ \qquad \qquad -{\mathcal {W}}_{{\mathcal {L}}}({u_{1}}_{\iota }-{\mathcal {F}}_{{\mathcal {L}}})+\Im +\frac{{\mathcal {W}}_{\epsilon }}{\sum \limits _{\jmath =1}^{{\textbf{N}}}{\tilde{c}}_{\iota \jmath }}\sum \limits _{\jmath =1}^{{\textbf{N}}}{\tilde{c}}_{\iota \jmath }({u_{1}}_{\jmath }-{u_{1}}_{\iota }),\\ \,^{c}\Delta _{{\textbf{a}}}^{\vartheta _{\iota }}{u_{2}}(\xi )=\varphi \cosh ({u_{1}}_{\iota }-{\mathcal {F}}_{3})/2{\mathcal {F}}_{4}\left( 1/2(1+\tanh (({u_{1}}_{\iota }-{\mathcal {F}}_{3})/{\mathcal {F}}_{4}))-{u_{2}}_{\iota }\right) ,\,\,\iota =1,2,\ldots ,{\textbf{N}},\end{array}\right. } \end{aligned}$$

where ${\mathcal {W}}_{e}>0$ denotes the system’s electrically powered interaction. The system’s connection matrix is expressed by ${\textbf{M}}=({\tilde{c}}_{\iota \jmath })_{{\textbf{N}}\times {\textbf{N}}}.$ Additionally, we separate a group of size ${\textbf{N}}$ into two particular groups based on fractional-order expressed as $\vartheta _{\iota }=\left[ \underbrace{\vartheta ,\ldots ,\vartheta }\limits _{{\textbf{r}}},\underbrace{\Phi ,\ldots ,\Phi }\limits _{{\textbf{s}}}\right] ,$ where ${\textbf{r}}$ components possess similar fractional-order, indicating fluctuating behaviour, while the rest of the ${\textbf{s}}$ points have fractional-order, indicating activated behaviour. Consequently, the general population capacity ${\textbf{N}}$ is given by ${\textbf{N}}={\textbf{r}}+{\textbf{s}}.$ We begin by investigating the behaviour of independently associated group **i** activated Morris–Lecar neural networks with two fractional-order factors, $\vartheta =1$ and $\Phi =0.80$. The entire amount of components in the system is ${\textbf{N}}=100,$ using ${\textbf{r}}=65$ and ${\textbf{s}}=35$ indicating that we are considering a system of neuronal populations with 65% oscillated neural networks and 35% activated nerve cells. In a condition without any association (${\mathcal {W}}_{\epsilon }=0.001$), every fluctuating nerve cell in the structure exhibits dominant exploding, while the additional nerve cells stay dormant. In addition to a negligible improvement in electrical power attachment ${\mathcal {W}}_{\epsilon }=0.0005$, the fluctuating subgroup continues to have dominant spikes in setting, while an alternate subdivision goes into quiescence. Red and blue lines denote the cumulative information of two independently interconnected components representing two particular groups (see Fig. 5a and b). The red line connection is picked at random based on the inactive components, and the blue line connection is compared to the increasing nodes in the network. The space-time plot shows that the spikes in components (1–65) are asymptotic (see Fig. 5e,f). When the relationship is increased 100 times (${\mathcal {W}}_{\epsilon }=0.05$), the framework’s behaviour alters. The previously dormant subgroup is presently exhibiting overflowing behaviour. In particular, the time time-frame between each wave of activity is not constant. An additional subgroup exhibits asymptotic unsteady active spiked (see Fig. 5i,j). It is obvious that as the degree of association in the transformed group increases, the sporadic and additionally dormant structure of the structure disappears, and inconsistent overflowing or jumping comes up. The neural network as a whole exhibits overflowing interactions via a limited number of surges during every explosion and a modest rise of ${\mathcal {W}}_{\epsilon }=0.007$ (see Fig. 5g,h). Two layers possess distinct intensities; however, identical stages develop in this particular instance. Subsequently, at ${\mathcal {W}}_{\epsilon }=1,$ the interconnected structure displays nearly synchronization practises by switching to active jumping (see Fig. 5k,l).

When we improve the momentary stimulation to $\Im =45,$ the fluctuating subgroup exhibits swift spikes in while another subsection keeps quiescent. At minimal attachment (${\mathcal {W}}_{\epsilon }=0.0005$), neither subgroup’s behaviour changes. These include specific groups that begin terminating and exhibit overflowing interactions such that the dependence ${\mathcal {W}}_{\epsilon }=0.005$ and 0.06 increases. Ultimately, at ${\mathcal {W}}_{\epsilon }=1,$ the sporadic network operation transforms into synchronized regular spikes (Fig. 5c,d). The group **ii** activated Morris–Lecar neural networks are then studied in an identical structure configuration, featuring a subgroup dormant ( $\Phi = 0.72$) and an additional showing mixed mode oscillations ($\vartheta =0.84$) in their dearth of interaction. Nerve cells are asynchronized when there is an inadequate connection (${\mathcal {W}}_{\epsilon }=0.0005,\,0.05$). As integrating increases, every link in the structure exhibits surge regularity modification (${\mathcal {W}}_{\epsilon }=0.05$ and ${\mathcal {W}}_{\epsilon }=1$). It is obvious that every one of the two separate arrays of oscillating elements demonstrates nearly perfect synchronization, via analogous steps and intensities (see Fig. 5).

### Incommensurate discrete fractional-order three-dimensional Morris–Lecar neural networks model

**Figure 6:**
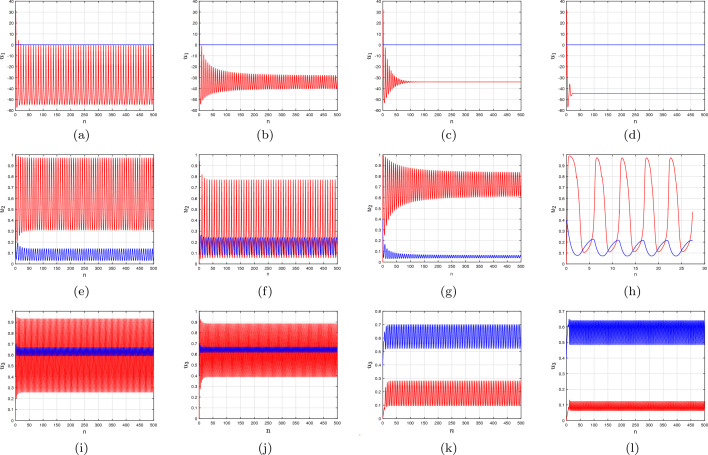
Nature of neural network reactions depending on association abilities in a reduction in order discrete fractional-order three-dimensional Morris–Lecar system (19) for activated cells of group **i** and group **ii** via multiple fractional-orders. (**a**–**d**) Group **i**: $(\vartheta ,\Phi ,\gamma )=(1,0.95,0.85)$ and ${\mathcal {W}}_{\epsilon }=0.0005,\,0.005,\,0.05,1,$ respectively. (**e**–**h**) Group **ii**: $(\vartheta ,\Phi ,\gamma )=(0.95,0.85,0.80)$ and ${\mathcal {W}}_{\epsilon }=0.0005,\,0.007,\,0.05,1.$ (**i**–**l**) Group **iii**: $(\vartheta ,\Phi ,\gamma )=(0.90,0.73,0.65)$ and ${\mathcal {W}}_{\epsilon }=0.0005,\,0.007,\,0.05,1$.

Here, a modified three-dimensional discrete fractional-order Morris–Lecar neural networks system is presented to demonstrate that the reduction in fractional-order analyses the identical characteristics exhibited by the executed arbitrary network techniques. We discovered an additional accumulation of synchronization status in the framework during the intermediary interaction. Inspired by this information, we are able to compose ${u_{1}}_{1}={u_{1}}_{2}={u_{1}}_{3}=\ldots ={u_{1}}_{60}={u_{1}}_{\vartheta }$ and ${u_{1}}_{61}={u_{1}}_{62}=\ldots ={u_{1}}_{100}={u_{1}}_{\Phi }.$ Given our knowledge of the Erdös–Rényi plot, one can estimate the level of every component or neural network by the standard extent of the system under consideration^52^. As a result, we are able to conclude that the pitch of the $\jmath$ cluster is $\eta _{\jmath }=\langle \eta \rangle .$ The total quantity of spiked oscillating elements in the vicinity for every oscillating device is anticipated to be $(1-\wp _{\epsilon })\eta =\wp _{0}\eta ,$ while the result associated with inactive oscillating elements will be $\wp _{\epsilon }\eta$. As a result, we can create a reduced-order simulation involving two oscillating elements, as shown below:

26 $$\begin{aligned} {\left\{ \begin{array}{ll} {\mathcal {C}}\,^{c}\Delta ^{\vartheta }{u_{1}}(\xi )=-1/2{\mathcal {W}}_{C_{a_{1}}}({u_{1}}_{\vartheta }-1)\left( (1+\tanh ({u_{1}}_{\vartheta }-{\mathcal {F}}_{1}))/{\mathcal {F}}_{2}\right) -{u_{2}}_{\vartheta } {\mathcal {W}}_{{\mathcal {K}}}({u_{1}}_{\vartheta }-{\mathcal {F}}_{{\mathcal {K}}})-{\mathcal {W}}_{{\mathcal {L}}}({u_{1}}_{\vartheta }-{\mathcal {F}}_{{\mathcal {L}}})\\ \qquad \qquad \qquad +\Im +{\mathcal {W}}_{\epsilon }\wp _{\epsilon }({u_{1}}_{\Phi }-{u_{1}}_{\vartheta })\\ \,^{c}\Delta ^{\vartheta }{u_{2}}(\xi )=\varphi \cosh \left( ({u_{1}}_{\vartheta }-{\mathcal {F}}_{2})/2{\mathcal {F}}_{4}\right) \left( 1/2(1+\tanh \left( {u_{1}}_{\vartheta }-{\mathcal {F}}_{3}\right) /{\mathcal {F}}_{4})-{u_{2}}_{\vartheta }\right) ,\\ \,^{c}\Delta ^{\vartheta }{u_{3}}(\xi )=\theta ({\mathcal {F}}_{0}+{u_{1}}_{\vartheta }),\\ {\mathcal {C}}\,^{c}\Delta ^{\Phi }{u_{1}}(\xi )=-1/2{\mathcal {W}}_{C_{a_{1}}}({u_{1}}_{\Phi }-1)\left( (1+\tanh ({u_{1}}_{\Phi }-{\mathcal {F}}_{1}))/{\mathcal {F}}_{2}\right) -{u_{2}}_{\Phi } {\mathcal {W}}_{{\mathcal {K}}}({u_{1}}_{\Phi }-{\mathcal {F}}_{{\mathcal {K}}})-{\mathcal {W}}_{{\mathcal {L}}}({u_{1}}_{\Phi }-{\mathcal {F}}_{{\mathcal {L}}})\\ \qquad \qquad \qquad +\Im +{\mathcal {W}}_{\epsilon }\wp _{0}({u_{1}}_{\vartheta }-{u_{1}}_{\Phi })\\ \,^{c}\Delta ^{\Phi }{u_{2}}(\xi )=\varphi \cosh \left( ({u_{1}}_{\Phi }-{\mathcal {F}}_{2})/2{\mathcal {F}}_{4}\right) \left( 1/2(1+\tanh \left( {u_{1}}_{\Phi }-{\mathcal {F}}_{3}\right) /{\mathcal {F}}_{4})-{u_{2}}_{\Phi }\right) ,\\ \,^{c}\Delta ^{\Phi }{u_{3}}(\xi )=\theta ({\mathcal {F}}_{0}+{u_{1}}_{\Phi }),\\ {\mathcal {C}}\,^{c}\Delta ^{\gamma }{u_{1}}(\xi )=-1/2{\mathcal {W}}_{C_{a_{1}}}({u_{1}}_{\gamma }-1)\left( (1+\tanh ({u_{1}}_{\gamma }-{\mathcal {F}}_{1}))/{\mathcal {F}}_{2}\right) -{u_{2}}_{\gamma } {\mathcal {W}}_{{\mathcal {K}}}({u_{1}}_{\gamma }-{\mathcal {F}}_{{\mathcal {K}}})-{\mathcal {W}}_{{\mathcal {L}}}({u_{1}}_{\gamma }-{\mathcal {F}}_{{\mathcal {L}}})\\ \qquad \qquad \qquad +\Im +{\mathcal {W}}_{\epsilon }\wp _{1}({u_{1}}_{\gamma }-{u_{1}}_{\vartheta })\\ \,^{c}\Delta ^{\gamma }{u_{2}}(\xi )=\varphi \cosh \left( ({u_{1}}_{\gamma }-{\mathcal {F}}_{2})/2{\mathcal {F}}_{4}\right) \left( 1/2(1+\tanh \left( {u_{1}}_{\gamma }-{\mathcal {F}}_{3}\right) /{\mathcal {F}}_{4})-{u_{2}}_{\gamma }\right) ,\\ \,^{c}\Delta ^{\gamma }{u_{3}}(\xi )=\theta ({\mathcal {F}}_{0}+{u_{1}}_{\gamma }), \end{array}\right. } \end{aligned}$$

where $\wp _{\epsilon }=\frac{{\textbf{s}}}{{\textbf{N}}},\,\wp _{0}=\frac{{\textbf{r}}}{{\textbf{n}}}$ and $\wp _{1}=\frac{{\textbf{x}}}{{\textbf{N}}}$ are the likelihoods of activated and fluctuating neurons in general. For group **i** and group **ii** Morris–Lecar scenarios, we operated computational experiments utilizing lower-order two-connected frameworks whereby every group is represented through an alike fractional-order exponent demonstrating concurrent behaviour. The computational findings indicate that whenever the two specific populations adhere to collection synchronization, the fluctuations of the reduced-order simulation obey the same structure as the entirety chart (see Fig. 6a–d). For example, overflowing involving two surges might occur for temporary connections in a group **i** activated framework detached by two layers in the entire system (see Fig. 6e–h). The minimized control system has an analogous bursting structure (see Fig. 6i–l). In the field of neuroscience, Hopf bifurcation occurs when neuron behaviours fluctuate from halting to spiking (the stable, steady options match the state of rest, and the spiking state demonstrates the presence of oscillating solutions).

A further typical form of dynamic activity for a neuron cell happens when, as the controlling value is increased, a saddle point and a limit cycle come together, resulting in a saddle-homoclinic bifurcation. The period of the periodic orbit that seems to be at the point of bifurcation approaches infinity, and as the controlling value is increased more, the periodic orbit disintegrates. It is shown that the formation and demise of saddle-homoclinic bifurcation in model (26) depend on implemented energy $\Im \in [23,70]$ (see Fig. 6), similar to the original model (3), but for fractional-order models of orders $\vartheta =1.0,\,0.95$ and 0.90,  the neuron requires greater applied current $\Im$ to bifurcate. When saddle homoclinic bifurcation occurs, neurological research predicts the occurrence or elimination of spiking practices (see Figs. 2 and 4).

## Conclusion

We started with the behaviour of just one neuron, which can evolve its reactions to different feedback data analyses contingent on the discrete fractional-order commensurate and incommensurate cases. We addressed the activities of groups **i** and **ii** by electrically conducting two-dimensional Morris–Lecar in pragmatic neural networks of commensurate and incommensurate order and reduced orders that are capable of being captured by experimentation. We demonstrated that variations in the discrete fractional-order and system settings influence the stipulates of the suggested approach, and we acquired an assortment of interactions involving steady pathways, regular actions and chaotic behaviours. We investigated the classical-order model’s associated bifurcation assessment. The discrete fractional-order may generate dismissing distinctions that are not visible in integer-order fluctuations. With the aid of a reduction in the discrete fractional-order scheme, the potential of the two-dimensional Morris–Lecar simulation for varied surge reactions involving mixed mode oscillations and mutated monarch butterfly optimizations via fractional difference is investigated. The findings show that the slow-fast three-dimensional Morris–Lecar in the fractional context produces a variety of packed sequences. Furthermore, it transforms its behaviour from overflowing into a sequence of rises in addition to swift spikes into inconsistent abundance depending on an appropriate collection of factors, which the classical-order simulation is unable to measure for a specific set of factors. The discrete fractional-order commensurate depicts a new illustration of the spiking and exploding reactions with fractional exponent transforms in the unpredictable representations. The method summarized the numerous responses of an individual-activated simulation to an assigned trigger variability using memory-contingent procedures. We examined the significance of electromagnetic connections in an indeterminate system with a subset of nodes in a dormant condition. The whole population would demonstrate rising regularity in adjusting if the fluctuating components were in mutated monarch butterfly optimizations. When the disconnected oscillated components persist in the rapidly active jumping region, all of the population splits into a pair of layers, demonstrating continuous overflowing in temporary association and active shooting in more substantial interaction. We were additionally capable of simplifying the network’s complexity into separate, complemented behaviours that effectively represented the changing patterns of the whole system throughout accumulation synchronization, thanks to the clustered synchronization phenomenon. Using the discrete fractional-order incommensurate, we discovered unique impacts on various membrane electrical attributes. Moreover, variations in neurological attributes depend on the memory consequences. The discrete fractional-order difference has potential interactions and can be used to investigate pragmatic occurrences. These findings show that the mathematical framework and networks present a comprehensible strategy for studying neurological behaviour.

## Data Availability

The datasets used and/or analyzed during the current study available from the corresponding author on reasonable request.
